# Searching for the Critical *p* of Macphail’s Null Hypothesis: The Contribution of Numerical Abilities of Fish

**DOI:** 10.3389/fpsyg.2020.00055

**Published:** 2020-01-31

**Authors:** Maria Elena Miletto Petrazzini, Alessandra Pecunioso, Marco Dadda, Christian Agrillo

**Affiliations:** ^1^School of Biological and Chemical Sciences, Queen Mary University of London, London, United Kingdom; ^2^Department of General Psychology, University of Padova, Padua, Italy; ^3^Padua Neuroscience Center, University of Padova, Padua, Italy

**Keywords:** fish, counting, non-symbolic numerical abilities, approximate number system, inter-species differences

## Abstract

In 1985, Macphail argued that there are no differences among the intellects of non-human vertebrates and that humans display unique cognitive skills because of language. Mathematical abilities represent one of the most sophisticated cognitive skills. While it is unquestionable that humans exhibit impressive mathematical skills associated with language, a large body of experimental evidence suggests that Macphail hypothesis must be refined in this field. In particular, the evidence that also small-brained organisms, such as fish, are capable of processing numerical information challenges the idea that humans display unique cognitive skills. Like humans, fish may take advantage of using continuous quantities (such as the area occupied by the objects) as proxy of number to select the larger/smaller group. Fish and humans also showed interesting similarities in the strategy adopted to learn a numerical rule. Collective intelligence in numerical estimation has been also observed in humans and guppies. However, numerical acuity in humans is considerably higher than that reported in any fish species investigated, suggesting that quantitative but not qualitative differences do exist between humans and fish. Lastly, while it is clear that contextual factors play an important role in the performance of numerical tasks, inter-species variability can be found also when different fish species were tested in comparable conditions, a fact that does not align with the null hypothesis of vertebrate intelligence. Taken together, we believe that the recent evidence of numerical abilities in fish call for a deeper reflection of Macphail’s hypothesis.

## Introduction

The capacity to process numerical information represents one of the most sophisticated cognitive skills in our species. Studies on individuals living in non-Western societies with a limited vocabulary for numbers showed that an adult human brain *per se* is not enough to elaborate complex mathematical skills. Culture and language play a fundamental role in developing abstract numerical competence (Dehaene et al., 2008). For instance, native speakers of Mundurukú have a limited vocabulary for numbers (only for the numbers 1 through 5). This Amazonian indigenous group proved to have an exact arithmetic with numbers smaller than 5. However, they are also able to compare and add large numbers far beyond their naming range, showing the existence of “non-symbolic” numerical abilities that are approximate and independent from language and culture (Pica et al., 2004). Apart from cross-cultural studies, developmental (Izard et al., 2009) and cognitive (Revkin et al., 2008) psychology also showed the existence of non-symbolic numerical abilities. These cognitive skills are supposed to be shared with other vertebrates (Feigenson et al., 2004; Beran, 2008). Rudimentary numerical abilities in animals have been reported since the 1930s, mainly mammals and birds (Koehler, 1941; Hauser et al., 1996; Brannon et al., 2001; Agrillo and Bisazza, 2018). Especially, the capacity to discriminate the larger/smaller group of biologically relevant items is supposed to solve most of the quantitative problems encountered in nature (e.g., select the most advantageous group of food items, sexual mates, or social companions).

The first evidence of numerical abilities in cold-blooded vertebrates was provided by Uller et al. (2003), studying amphibians. Since then, we have witnessed an increase in the publications on this group of vertebrates, mainly represented by studies on fish (reviewed in Agrillo and Bisazza, 2018). The discovery that small-brained species that lack cortex, such as fish, display similar numerical abilities described in humans represents a true challenge to the hypothesis advanced by Macphail (1985). In his seminal paper, the author argued that *there are neither quantitative nor qualitative differences among intellects of non-human vertebrates* (p. 37). Also, he claimed that *man’s intellectual superiority may be due solely to our possession of a species-specific language-acquisition device* (p. 37). Any evidence in support of a surprising similarity in numerical abilities of humans and fish would be an argumentation against the humans’ superiority of cognitive skills advanced by Macphail (1985). Indeed, fish represent the vertebrate group more distantly related to humans, as fish and land vertebrates diverged approximately 450 million years ago. The structure of the brain is largely different in terms of size and neural circuits. Besides these aspects, the aquatic environment is clearly incomparable with the dry land occupied by primates (and most mammals in general), a fact that is likely to have differently impacted on the selective pressures that shaped cognitive skills. Lastly, fish represent approximately half of vertebrate species. Most of these species occupy very different ecological niches, ranging from dense environments of shallow waters of the rivers to empty environments in the deep waters of the oceans. In this sense, they represent the ideal vertebrate group to study the existence of interspecies differences, a fact that would contrast with the null hypothesis of vertebrate intelligence.

In this work, we review the literature of numerical abilities of fish analyzed under the prism of Macphail’s argumentations. The first part of the work will be devoted to outlining the evidence against the null hypothesis; the second part will summarize why we should not reject this hypothesis. Lastly, we will suggest some future directions necessary to form a broader comprehension in this field.

## Rejecting the Null Hypothesis (*p* < 0.05)

In this section, we will split our argumentations in two main directions, starting from the statements made by Macphail in 1985: the absence of difference in cognitive abilities of animals and the superiority of humans’ intellectual skills.

### Neither Quantitative Nor Qualitative Differences Among Vertebrates

According to Macphail, similar cognitive abilities among the species are expected. However, data coming from numerical cognition studies in fish do not support this view. There is evidence that numerical acuity is different across the species. This is clear, for instance, in the different ability of teleost fish to select the larger shoal when exploring a novel and potentially dangerous environment. Such ability is supposed to be highly useful in nature to reduce the risks of being predated. It has been shown that the capacity to discriminate between a large and a small shoal varies as a function of the species: when the two shoals differ by one unit, angelfish seem to be able to find the larger shoal up to 3 units (2 vs. 3, Gómez-Laplaza and Gerlai, 2011), mosquitofish up to 4 (3 vs. 4, Agrillo et al., 2008), guppies up to 5 (4 vs. 5, Lucon-Xiccato et al., 2017), while stickleback seem to be able to discriminate even 6 from 7 conspecifics (Mehlis et al., 2015). As these species are highly social, it is unlikely that the variability here observed could be explained by the different degree of motivation in reaching social companions. Also, one may argue that such differences are the results of different stimuli and procedures. There is indeed evidence that the precision in numerical tasks is affected by the experimental procedure adopted (Gatto et al., 2017). For instance, the capacity to discriminate two vs. three social companions in goldbelly topminnows depends on the type of stimulus presentation (two shoals presented on the same side of the tank vs. two shoals presented on the opposite sides of the tank, Agrillo and Dadda, 2007). This is exactly what Macphail (1985) was referring to about the difficulty to establish if the different performance reported among vertebrates actually reflects true inter-species differences in cognitive skills or instead reflects the consequence of contextual variables, such as the type of methodology used.

For this reason, a fine comparative study of the numerical ability of animals should take into account this issue, reducing the methodological variability among the species. To tackle this problem, Agrillo et al. (2012a) tested numerical acuity of five different teleost fish using the same stimuli, apparatus, and procedure. Two sets of two-dimensional figures of different numerosities were presented at the opposite ends. Food was provided only near the stimulus to be reinforced. The proportion of time spent near the positive stimulus in probe trials without food reward was used as a dependent variable. This training procedure was applied to five different fish species: redtail splitfin (*Xenotoca eiseni*), guppies (*Poecilia reticulata*), zebrafish (*Danio rerio*), angelfish (*Pterophyllum scalare*), and Siamese fighting fish (*Betta splendens*). The same visual patterns were presented to all subjects. In one experiment, subjects were initially trained on a 0.5 ratio (5 vs. 10 and 6 vs. 12 figures). For instance, they were required to select the larger array to receive a food reward. Once they reached the learning criterion, they were presented with novel numerical contrasts with harder numerical ratios: 0.67 (8 vs. 12) and 0.75 (9 vs. 12). In another experiment, after reaching the learning criterion, they were observed in their capacity to generalize the numerical rule to very small (2 vs. 4) or very large (25 vs. 50) numerical contrasts. Overall, fish proved able to generalize the learned rule to harder numerical contrasts (0.67 ratio but not 0.75) and were able to generalize it to a smaller set of items (2 vs. 4 but not 25 vs. 50). However, a deeper analysis of fish performance suggested at least two main inter-specific differences: angelfish was not able to discriminate between 8 and 12 items, suggesting a lower numerical acuity. Similarly, the performance of zebrafish was lower in terms of proportion of individuals that reached the learning criterion. Although alternative explanations were also taken into account by the authors, these two results leave open the concrete possibility that quantitative differences exist in the cognitive processes underlying numerical estimation of fish. This hypothesis is further supported by a study on a blind cavefish (*Phreatichthys andruzzii*) that evolved for approximately 2 million years in the phreatic layer of the Somalia desert (Bisazza et al., 2014a). As they lack visual modality, the training procedure adopted in the previous inter-specific study was partially modified to include three-dimensional stimuli submerged in the tank (instead of two-dimensional figures). The subjects were trained to discriminate between two groups of sticks placed in opposite positions of the experimental tank in order to receive a food reward. Cavefish showed the ability to discriminate accurately two vs. four objects but not two vs. three. This indicates that the brains of fish species that live in a very peculiar ecological niche (dark caves with no predators) are still equipped with neural circuits that support numerical processing. However, it is worth noting that cavefish showed lower performance in terms of numerical acuity compared to the majority of fish species investigated (that commonly discriminate 0.67 ratio, e.g., Agrillo et al., 2012a,b). At least three main hypotheses have been advanced: Provided that cavefish cannot use a visual modality to solve these tasks, the most likely explanation is that they used the lateral line, a sense organ typical of fish, which is integral to detecting movement, vibration, and pressure gradients in water. It is possible that object representation through lateral line might be less precise. If so, cavefish might have the same numerical acuity of other species but exhibit a worse performance because of a general noise in detecting the items to be enumerated. Another possibility is that cognitive numerical systems might be more accurate in the visual modality. Tokita et al. (2013) found in human participants a different performance in numerosity judgments tested in visual and auditory conditions, advancing the idea of multiple numerical systems—with different degrees of precision—related to the different sensory modalities. Lastly, it is possible that the peculiarity of the ecological niche plays an important role in shaping numerical systems. This species evolved for millions of years in a homogeneous environment with a scarcity of food resources and without natural predators. Selective pressures might have acted reducing the cerebral mass in order to optimize the metabolic consumption of the brain, thus lessening also the neural circuits supporting cognitive functions not useful in a cave’s life.

In sum, the comparative investigation of fish species tested with reduced methodological variability (similar apparatuses, stimuli, and procedures) provided enough experimental material to argue that the assumption of no inter-specific differences among the species can be hardly sustained, at least with respect to numerical cognition.

### Man’s Intellectual Superiority

Humans are clearly very precise in numerical tasks compared to fish (and presumably to all other animals, see Section “Failing to Reject the Null Hypothesis (*p* > 0.05)”). However, if a superiority does exist in absolute terms, it is expected to emerge also in issues other than numerical acuities, such as the cognitive mechanisms used to estimate quantities.

It is known than numerosity co-varies with several other physical attributes of the stimuli, also known as “continuous quantities,” such as cumulative surface area (i.e., the sum of areas of the items to be enumerated), density, and convex hull (the overall space occupied by the most lateral items of the array). There is evidence that humans involved in non-symbolic numerical tasks can establish which group of objects is larger by using a combination of discrete (numerical) and continuous information (Gebuis and Reynvoet, 2012a,b; see Leibovich et al., 2017 for a review about). In short, when comparing two groups of three and four circles, we would extrapolate both the numerosity of items and the associated continuous quantities. The capacity to discriminate the larger/smaller group would be the result of this number-space interplay.

There is evidence that also fish can process both numerical and continuous quantities. A decade ago, Agrillo et al. (2009) provided the first evidence that fish can use numerical information also when all continuous quantities were controlled for. Mosquitofish (*Gambusia holbrooki*) were placed in an unfamiliar environment. To re-join their social companions, subjects were required to select one of two identical tunnels at opposite corners. The correct tunnel was associated with a specific number of items (either two or three) presented above the tunnels. The shapes and spatial arrangements of the figures were changed across the trials to prevent the fish from learning to recognize specific patterns. Furthermore, the items were controlled for continuous quantities so that the only discriminative cue was numerical information. Subjects proved able to solve the task, indicating the use of numerical information by fish. To date, we know that at least eight fish species can process numerical information in different experimental contexts (Agrillo and Bisazza, 2018).

Fish, however, can also use continuous quantities. Agrillo et al. (2009) set up an experiment in which the fish were trained to discriminate between two and three figures in a condition in which the number and continuous quantities were simultaneously available. For example, the larger group occupied also the larger area. In the test phase, researchers controlled for one continuous quantity at a time and observed the performance of mosquitofish: accuracy decreased when the stimuli were matched for the cumulative surface area or the convex hull, indicating that these cues had been used during the tasks. The combination of these continuous quantities is exactly what has been advanced as an important mechanism for human numerical estimation. According to the occupancy model (Allik and Tuulmets, 1991), numerosity estimation is linearly related to the total area occupied (occupancy) by virtual disks that circumscribe each dot. When dots are close to one other, the virtual disks overlap, leading to an underestimation of the dots; when the dots are more distant, the overall space occupied by these disks is larger, leading to an overestimation of the dots. Therefore, the combination of cumulative surface area and inter-item distance (a parameter that is linearly related to the convex hull) seems to influence numerical estimation of both humans and mosquitofish.

The use of discrete and continuous quantities has been reported not only in the presence of neutral laboratory stimuli (such as two-dimensional stimuli) but also with biologically relevant stimuli. For what concern discrete information, Dadda et al. (2009) found that mosquitofish can select the larger shoal also when stimulus fish were presented one at a time and hence are required to sum the number of fish contained in each shoal. Similar capacity was later observed in newborn guppies (Bisazza et al., 2010), suggesting the existence of inborn numerical abilities in fish similar to that described in human infants (Izard et al., 2009). Continuous quantity discrimination in a highly ecological context was studied by Lucon-Xiccato et al. (2015). The authors found that guppies are able to select the larger piece of food when the ratio between the smaller and larger piece is 0.75.

Clustering is another perceptual cue that affects non-symbolic numerical tasks. Humans tend to overestimate the number of items if they are arranged to form a single Gestalt. This is particularly evident in the Solitaire illusion studied by Frith and Frith (1972), a visual pattern in which items forming a single Gestalt is overestimated compared to the same number of items arranged in separate (smaller) clusters. Perception of the Solitaire illusion has been recently studied in fish. Guppies were trained to select an array containing a larger quantity of black dots in the presence of two arrays made by white and black dots. After reaching the learning criterion, subjects were presented with two illusory arrangements: One array presented 16 black dots centrally located to form a single Gestalt and 16 white dots on the perimeter to form 4 separate clusters; the other presented 16 white dots centrally located with 16 black dots on the perimeter. If the subjects perceived the illusion, they were expected to select the array in which the black dots were centrally located (as they appear to be larger to human observers). Although higher inter-individual variability was found in fish compared to humans (Agrillo et al., 2016; Pecunioso and Agrillo, 2019), guppies exhibited a human-like susceptibility to this numerosity illusion, suggesting that clustering of items is a further common mechanism used by both humans and fish to estimate the number of items in the visual scene.

It is important to clarify that humans appear to be equally able to use numerical information over continuous quantities (Hurewitz et al., 2006). One may argue that animals might find it more difficult to process numerical information than continuous quantities. This was indeed the idea advanced by different authors in the 1980s (Davis and Memmott, 1982; Davis and Perusse, 1988) that led to the hypothesis of numerical information as “last-resort strategy” used only when no other continuous quantity would permit an animal to discriminate which group is larger/smaller. A study by Agrillo et al. (2011) does not encourage to this view. Three groups of mosquitofish were trained in different conditions: In one condition, the mosquitofish could use only numerical information to distinguish between the quantities (2 vs. 3, “numerical” condition). In the second condition, fish could use only continuous quantities (1 vs. 1, the ratio between the areas was equal to two-thirds, the “continuous quantity” condition). In the third condition, both numerical and continuous information was available (2 vs. 3, with the larger group occupying more space, “number and continuous quantity” condition). If numerical information were more cognitively demanding, subjects were expected to need more trials to learn the task in the first condition than in the other two conditions. As expected, higher performance was found when fish could use both numerical and continuous quantities as the presence of multiple cues is supposed to represent the easiest (and the most ecological) condition (Gebuis and Reynvoet, 2012a,b). However, no difference was found between the numerical condition and the continuous quantity condition, suggesting that, at least for mosquitofish, processing numbers is not more complex than processing continuous quantities. After all, artificial neural networks suggest that numerosity estimation does not enroll a large neural network Hope et al. (2010), found that fewer than 25 units might be enough for a system to represent quantity with a performance comparable to that observed in fish (Agrillo et al., 2008). This is also supported by a more recent study (Stoianov and Zorzi, 2012), showing that as few as 35 hidden neurons were able to spontaneously extract numerical information in a visual scene. In this sense, it is not surprising that also a fish brain can apparently use number with the same cognitive effort used in continuous quantity discrimination.

The cognitive strategy used to learn a numerical rule is a further aspect that must be taken into consideration to establish similarities and differences between humans and fish. It is known that, when animals learn to select the larger of two arrays (e.g., 5 vs. 10), they might potentially use two alternative strategies. One strategy consists in learning to always select the array containing 10 items (“absolute numerical rule”). The other strategy consists of assessing which group is larger and smaller in order to “select the larger numerosity of each stimulus pair” (“relative numerical rule”). Because the behavioral output is the same, the exact cognitive strategy used by animals is often neglected. Miletto Petrazzini et al. (2016) dissociated the two hypotheses by training angelfish to discriminate between two arrays of figures differing in numerosity. One group of subjects was required to select 10 items in a 5 vs. 10 discrimination; the other group were required to select 10 items in the 10 vs. 20 discrimination. After reaching the learning criterion, the former group was presented with a 10 vs. 20 numerical contrast. If subjects had learned the task by using a relative numerical rule, they should have selected the novel larger numerosity (20); otherwise, if angelfish had used an absolute numerical rule, they were expected to select the numerosity previously reinforced (10). The other experimental group (10 vs. 20) was presented in test trials with 5 vs. 10 discrimination. Angelfish belonging to both groups spontaneously used a relative numerical rule, selecting the novel numerosity instead of the previously reinforced numerosity.

Interestingly, the authors also tested undergraduate students in the same task (Miletto Petrazzini et al., 2016). In order to observe the spontaneous use of a relative vs. absolute numerical rule, no verbal instructions were provided so that participants had to infer the numerical rule only by the feedback, exactly like fish. Humans used a relative numerosity rule too with very limited inter-individual variability. This implies that distantly related species share similar cognitive systems for making decisions about quantities, a fact that does not properly align with the idea of any kind of human’s superiority in terms of qualitative differences.

The similarities between humans and fish are not confined to the performance of the two species individually tested in cognitive tasks. It is known that interacting people can generally achieve more accurate decisions than single individuals. Although this is an open debate (e.g., Cantlon et al., 2006), it was suggested to occur also in numerical tasks. In a study by Bahrami et al. (2013), pairs of participants made both individual and collective estimations of which group of dots was larger. In the “collective enumeration” condition, they could negotiate joint decisions *via* verbal communication and received feedback about accuracy at the end of each trial. Results showed that two individuals collectively estimate the number of dots better than either one alone. Collective intelligence in non-human animals has been reported in different fields (Krause et al., 2010). However, although several species showed impressive numerical skills, including invertebrates (e.g., eusocial ants: Reznikova and Ryabko, 2011; Reznikova, 2017; bees: Pahl et al., 2013), no evidence of an advantage in collective enumeration was reported in non-human animals before 2014. Bisazza et al. (2014b) investigated this issue in fish. Guppies were observed in their spontaneous preference of joining the larger shoal (exp. 1) and in their capacity to learn a numerical rule after operant conditioning (exp. 2). Subjects’ performance was observed both when they were singly inserted in the experimental apparatus and when they were inserted in pairs. In both experiments, interacting guppies achieved a superior level of numerosity discrimination compared to the average ability of the isolated individual fish. Even though the reasons underlying the enhanced cognitive performance of interacting guppies are unknown, the result is intriguing as it suggests that the well-known collective intelligence that has been advanced in humans (Bahrami et al., 2013) can be traced also in a fish species.

In summary, we believe that all the above-mentioned studies provide a robust argumentation to say that the concept of *Man’s intellectual superiority* need to be deeply revised.

## Failing to Reject the Null Hypothesis (*p* > 0.05)

Here we will delineate why we believe that Macphail’s argumentation still holds in numerical cognition.

### Man’s Intellectual Superiority

As said in the “Introduction” section, it is unquestionable that the capacity of humans to process numerical information represents a clear example of high cognitive functions related to a species-specific language-acquisition device. However, we also display numerical abilities that are not related to language, a cognitive skill particularly evident when we are forced to estimate which group of objects is more numerous without the possibility to see the two groups long enough to count the objects (e.g., only 150–250 ms; Halberda et al., 2008; Revkin et al., 2008).

The comparison of non-symbolic numerical abilities of humans and animals clearly indicates that humans are more precise even in this numerical skill. Humans can discriminate a 0.90 ratio (9 vs. 10, Halberda et al., 2008), while numerical acuity of other species is often more limited (Hauser et al., 1996; Uller et al., 2003; Rugani et al., 2008). It is interesting to note that, although the superiority in numerical acuity of humans supports the null hypothesis, it also contradicts one of the predictions related to the importance of language: *Humans without language would, according to this view, be no more intelligent than non-human vertebrates* (Macphail, 1985, p. 49). Indeed, because non-symbolic numerical tasks prevent the use of verbal counting, one should not expect a higher performance of humans in this task. To tell the true story, participants involved in the studies that showed impressive abilities in non-symbolic numerical tasks were teenagers or university students of Western societies (e.g., Halberda et al., 2008; Agrillo et al., 2014). Even though experimental strategies were taken to limit the use verbal language, we cannot exclude that language and education of subjects positively impacted on the cognitive skills necessary to support numerical estimation, thus improving their performance. In line with this hypothesis, when members of non-Western societies are tested (e.g., Mundurukù: Pica et al., 2004; Warlpiri, and Anindilyakwa: Butterworth et al., 2008), participants’ performance in numerical estimation tasks is not far from that observed with several non-human species.

As said in “Rejecting the Null Hypothesis (*p* < 0.05)” Section, the observation of spontaneous behavior showed that sticklebacks can discriminate up to six vs. seven social companions (Mehlis et al., 2015). Other fish species, however, showed a lower performance when the groups to be compared differ by one unit (Agrillo et al., 2012c; Agrillo and Bisazza, 2018); therefore, the high level of performance exhibited by sticklebacks is not likely to reflect the average precision of fish in quantitative tasks. Trained fish guppies can also reach surprising performances (e.g., the capacity to discriminate up to 0.75 ratio, Bisazza et al., 2014c; Miletto Petrazzini et al., 2015a), but no study showed the capacity to discriminate up to a 0.90 ratio.

Humans’ superiority in numerical tasks extends far beyond relative numerosity judgments. Ordinal abilities are the capacity to understand that “3″ is larger than “2″ and smaller than “4.” This ability permits to solve several numerical tasks, including the capacity to locate an object on the basis of its position in a sequence of other objects. Unlike humans, fish showed a very limited ability to use ordinal information. Miletto Petrazzini et al. (2015b) trained guppies to select the third feeder in a row of eight alternative feeders placed perpendicularly in front of them. The inter-feeder distance was experimentally manipulated between trials to avoid the use of continuous quantities, such as the overall distance necessary to reach the correct feeder. The guppies solved the task, thereby providing the first evidence that ordinal abilities exist in fish species. However, in another experiment, researchers placed the correct feeder in the fifth position: In this case, the performance was no longer significant, showing a clear limit in using ordering information that does not exceed 3–4 units.

As said, the literature on fish in this section is just an example, since humans outperform mammals, birds, amphibians, reptiles, and fish in non-symbolic numerical tasks. All this literature clearly indicates that, although animals have recently shown evidence of impressive numerical abilities to demonstrate no qualitative differences, quantitative differences seem to exist between humans and animals.

## Conclusions and Future Remarks

We reviewed the literature on fish numerical cognition as a tool to shed light on the modernity of Macphail’s argumentations. We believe that most of the evidence collected in numerical tasks of fish call for a deep reframing of the null hypothesis of vertebrate intelligence. At least two different bodies of experimental evidence support our dissertation: (1) it is not true that numerical abilities in fish did not differ among the species and (2) most of the literature speaks in favor of qualitative similarities between humans and fish. Evidence supporting the first claim comes from studies in which numerical abilities of fish were compared with the same experimental material/procedure. These studies showed that, although similarities are greater than differences, inter-species differences exist among fish. The latter claim is supported by studies showing a similar use of discrete and continuous quantities in human and fish, by the observation of comparable cognitive strategy to learn a numerical rule and by the evidence of an enhanced numerical performance when multiple individuals are involved in the numerical task.

However, rejecting this hypothesis might be precocious at this stage. The null hypothesis of Macphail still holds if one considers a crucial aspect of numerical abilities, the precision of numerical estimation. Although in the last two decades several studies showed impressive numerical abilities in fish (and in animals in general), the higher performance is repeatedly reported in humans, even in tasks in which they are prevented to use verbal counting. Therefore, if any clear difference exists between human and fish, such difference is quantitative but not qualitative.

That said, it is important to specify that the procedure in human and animal studies often differs for a fundamental aspect: The presence/absence of verbal instructions. As known, animals have to infer the rule trial by trial, while most of human studies are often introduced by verbal instructions (Halberda et al., 2008; Revkin et al., 2008; Price et al., 2012). This permits participants to focus on the most relevant aspects of the experiment since the beginning, providing a potential advantage that might be misinterpreted as higher numerical abilities. Only recently researchers have begun to take into account this potential confound and present human participants with tasks with no verbal instructions (Beran, 2006; Miletto Petrazzini et al., 2016; Parrish et al., 2019).

Some important issues need to be investigated. To better understand the similarities between humans and fish it would be important to assess whether fish display an abstract concept of number. We know that humans can compare quantities of objects presented in different sensory modalities (e.g., three lights and three sounds). The capacity to transfer numerical information from the visual to the acoustic modality is important evidence of an abstract concept of number. To date, existing studies in fish reported the capacity to generalize the numerical rule to novel stimuli presented through the same sensory modality (e.g., visual stimuli; reviewed in Agrillo et al., 2017). No study has established whether fish can transfer numerical information from one sensory modality to another, a fact that prevents to understand whether the complexity of abstract numerical representation is similar or not in human and fish. Also, the investigation of continuous quantities used by fish is limited to a few species (Agrillo et al., 2009, 2010, 2011; Gómez-Laplaza and Gerlai, 2013; Miletto Petrazzini et al., 2018). In order to understand whether the cognitive mechanisms used by human and fish are similar, we need to enlarge the number of species under investigation. Lastly, the spatial representation of numbers is another important aspect that should be considered to comprehend whether fish have human-like mechanisms of number processing. It is known that most humans represent numbers aligned from left to right, the so-called “mental number line” (Galton, 1880; Zorzi et al., 2002). There is evidence that also birds have a similar spatial representation of numbers (Rugani et al., 2015), but this issue has never been investigated in fish.

In 1985 Macphail said, *In common with all scientific hypotheses, this null hypothesis cannot be proved, only disproved; support for the hypothesis will grow as the number of failures to disprove it increases* (p. 46). After more than three decades, it is still difficult to reach a verdict on the hypothesis advanced by Macphail. We believe, however, that Macphail adopted a questionable statistical approach: when he introduced the idea of a “null hypothesis,” he indirectly assumed an all-or-none approach to this issue, like the frequentist *p* approach based on rejecting/failing to reject the null hypothesis. However, this statistical approach can barely grasp all the shades of this issue. For instance, how much do fish differ from other vertebrates, humans included, in numerical skills? If we look at numerical acuity of humans and fish, we would be inclined to assume that the null hypothesis is correct; if we look at qualitative similarities among the species we would be tempted to reject this null hypothesis. Is the null hypothesis corroborated or not? Instead of assuming that a dichotomic response may exist in this issue, we believe that the Bayesian approach would be more appropriate. Bayes factors actually enable researchers to estimate the relative strength of the evidence for two competing hypotheses (Dienes, 2014). Even supposing that the next decade will be characterized by the development of finer comparative methodologies, we believe that researchers, at best, could try to establish how likely is the null hypothesis of vertebrate intelligence over the alternative one.

## Author Contributions

All authors listed have made a substantial, direct and intellectual contribution to the work, and approved it for publication.

### Conflict of Interest

The authors declare that the research was conducted in the absence of any commercial or financial relationships that could be construed as a potential conflict of interest.
