# Corrigendum: Searching for the Critical *p* of Macphail's Null Hypothesis: The Contribution of Numerical Abilities of Fish

**DOI:** 10.3389/fpsyg.2020.01768

**Published:** 2020-08-06

**Authors:** Maria Elena Miletto Petrazzini, Alessandra Pecunioso, Marco Dadda, Christian Agrillo

**Affiliations:** ^1^School of Biological and Chemical Sciences, Queen Mary University of London, London, United Kingdom; ^2^Department of General Psychology, University of Padova, Padua, Italy; ^3^Padua Neuroscience Center, University of Padova, Padua, Italy

**Keywords:** fish, counting, non-symbolic numerical abilities, approximate number system, inter-species differences

There is an error in the Funding statement. The correct number for Marie Skłodowska-Curie Individual Fellowship from the European Union's Horizon 2020 is 750200.

The authors apologize for this error and state that this does not change the scientific conclusions of the article in any way. The original article has been updated.

